# Supplementary material for: De novo peptide databases enable protein-based stable isotope probing of microbial communities with up to species-level resolution
Source: Environ Microbiome. 2025 Aug 26;20:111. doi: 10.1186/s40793-025-00767-6 (PMC12379467; doi:10.1186/s40793-025-00767-6)
Supplement: Supplementary file 3 — Supplementary Material 3 [file 40793_2025_767_MOESM3_ESM.docx]

# Supplementary Material

Supplementary Table S1: Sizes of the de novo peptide databases employed in this study, with the number of peptides matching the corresponding genome-derived reference protein sequence database indicated in parentheses. The genome-derived reference protein sequence databases were appended with universal contaminants and comprised in total 123,365 protein sequences for dataset PXD024174, 5,344 for PXD057215, and 213,931 for PXD024291.

| **Database size = number of non-redundant *de novo* identified peptides** | | | | | | |
| --- | --- | --- | --- | --- | --- | --- |
| **Dataset identifier** | **PXD024174**  **(Mock community)** | | **PXD057215**  **(Anammox reactor)** | | **PXD024291**  **(Human gut model)** | |
| **Score threshold** | Casanovo | PepNet | Casanovo | PepNet | Casanovo | PepNet |
| unfiltered | 290,680  (23,675) | 875,366  (29,429) | 256,559  (7,521) | 303,595  (4,837) | 512,680  (59,600) | 715,031  (43,350) |
| 0.2 | 149,626  (22,743) | 258,054  (23,642) | 82,754  (6,897) | 33,867  (4,773) | 250,323  (55,807) | 225,748  (42,590) |
| 0.4 | 148,847  (22,730) | 175,840  (21,162) | 74,423  (6,896) | 23,321  (4,688) | 247,553  (55,799) | 170,283  (40,904) |
| 0.6 | 139,704  (22,689) | 119,472  (19,003) | 54,101  (6,882) | 16,636  (4,542) | 232,755  (55,705) | 128,653  (39,166) |
| 0.8 | 96,835  (22,426) | 80,093  (17,068) | 29,304  (6,852) | 12,001  (4,370) | 180,762  (55,242) | 97,990  (37,152) |
| 0.85 | 76,673  (22,097) | 71,180  (16,525) | 22,380  (6,781) | 10,960  (4,313) | 153,699  (54,693) | 90,692  (36,575) |
| 0.9 | 53,981  (21,137) | 61,634  (15,894) | 15,785  (6,562) | 9,819  (4,225) | 120,752  (53,113) | 82,718  (35,832) |
| 0.95 | 31,873  (18,813) | 50,450  (15,062) | 9,934  (6,034) | 8,473  (4,100) | 84,176  (48,997) | 72,797  (34,711) |
| 0.99 | 14,700  (13,156) | 35,375  (13,537) | 5,313  (4,644) | 6,605  (3,836) | 48,715  (38,054) | 58,476  (32,424) |

Supplementary Table S2: Number of taxon-specific peptides identified by database search via MS-GF+ using the CS>0.99 database in the PXD024174 dataset.

Supplementary Table S3: Drop-off data from *Escherichia coli*.

| **Labeled peptides** | **Total submissions** | **Root** | **Domain** | **Phylum** | **Class** | **Order** | **Family** | **Genus** | **Species** |
| --- | --- | --- | --- | --- | --- | --- | --- | --- | --- |
| in silico | 3,500 | 3,500 | 1,344 | 1,123 | 1,071 | 779 | 659 | 44 | 29 |
| main | 579 | 532 | 190 | 153 | 140 | 85 | 64 | 2 | 2 |
| others |  |  | 0 | 1 | 7 | 9 | 7 | 9 | 6 |
| random_main |  |  | 3 | 1 | 0 | 0 | 0 | 0 | 0 |
| random_others |  |  | 8 | 8 | 9 | 8 | 8 | 6 | 6 |
| main-random_main |  |  | 187 | 152 | 140 | 85 | 64 | 2 | 2 |
| others-random_others |  |  | -8 | -7 | -2 | 1 | -1 | 3 | 0 |
| purity [%] |  |  | 104.5 | 104.8 | 101.4 | 98.8 | 101.6 | 40.0 | 100.0 |
| main-random_main normalised |  |  | 100.0 | 81.3 | 74.9 | 45.5 | 34.2 | 1.1 | 1.1 |
| *in silico* normalised |  |  | 100.0 | 83.6 | 79.7 | 58.0 | 49.0 | 3.3 | 2.2 |
| drop off delta [%] |  |  |  | 2.8 | 6.4 | 27.5 | 43.3 | 206.1 | 101.7 |

Supplementary Table S4: Comparison of the number of detected ‘*Candidatus* Kuenenia stuttgartiensis’ strain CSTR1 peptides in unmixed and mixed samples of case study 1.

| **x hydraulic retention time** | | **0** | **1** | | **5.25** | |
| --- | --- | --- | --- | --- | --- | --- |
|  |  |  | **unmixed** | **mixed** | **unmixed** | **mixed** |
| ‘*Candidatus* Kuenenia stuttgartiensis’ strain CSTR1 peptides identified by MetaProSIP with genome-derived protein database | | 5,830 | 3,088 | 4,449 | 130 | 3,329 |
| ‘*Candidatus* Kuenenia stuttgartiensis’ strain CSTR1 peptides identified by *de novo* sequencing | Casanovo | 6,695 | 3,628 | 5,120 | 322 | 3,713 |
|  | PepNet | 4,336 | 2,035 | 3,171 | 97 | 2,131 |

Supplementary Table S5: Drop-off data from ‘*Candidatus* Kuenenia stuttgartiensis’.

| **Labeled peptides** | **Total submissions** | **Root** | **Domain** | **Phylum** | **Class** | **Order** | **Family** | **Genus** | **Species** |
| --- | --- | --- | --- | --- | --- | --- | --- | --- | --- |
| in silico | 3,500 | 3,500 | 2,544 | 2,326 | 2,312 | 2,312 | 2,258 | 2,081 | 2,081 |
| main | 1,290 | 1,225 | 894 | 814 | 811 | 811 | 790 | 669 | 669 |
| others |  | 0 | 0 | 1 | 1 | 1 | 1 | 1 | 1 |
| random_main |  | 339 | 27 | 0 | 0 | 0 | 0 | 0 | 0 |
| random_others |  | 0 | 35 | 40 | 40 | 37 | 37 | 35 | 32 |
| main-random_main |  | 886 | 867 | 814 | 811 | 811 | 790 | 669 | 669 |
| others-random_others |  | 0 | -35 | -39 | -39 | -36 | -36 | -34 | -31 |
| purity [%] |  | 100.0 | 104.2 | 105.0 | 105.1 | 104.6 | 104.8 | 105.4 | 104.9 |
| main-random_main normalised |  |  | 100.0 | 93.9 | 93.5 | 93.5 | 91.1 | 77.2 | 77.2 |
| *in silico* normalised |  |  | 100.0 | 91.4 | 90.9 | 90.9 | 88.8 | 81.8 | 81.8 |
| drop off delta [%] |  |  | 0.0 | 2.6 | 2.8 | 2.8 | 2.6 | 6.0 | 6.0 |

Supplementary Table S6: Relative isotope abundance of peptides assigned to gene ontology (GO) terms of biological processes related to subterms of amino acid metabolic processes (GO:0006520), carbohydrate derivative metabolic processes (GO:1901135), and carbohydrate metabolic processes (GO:0005975). P-values were calculated by Student's t-test for the means of two independent samples.

Supplementary Table S7: MS-GF+ runtime comparison between using *de novo* peptide databases (derived from Casanovo after filtering peptides with a quality score threshold > 0.99) and genome-derived protein databases. MS-GF+ was run on the UFZ Galaxy instance using 1 CPU and 57 GB RAM. While genome-derived protein databases were searched with semi-tryptic digestion enabled, *de novo* peptide databases were searched without any digestion enabled. Database size is given in megabytes.

| **Dataset identifier** | **Genome-derived protein database** | | **CS>0.99 peptide database** | |
| --- | --- | --- | --- | --- |
|  | **Database size [MB]** | **Runtime per sample [h]** | **Database size [MB]** | **Runtime per sample [h]** |
| PXD024174 (Mock community) | 51.8 | 53.5 | 0.4 | 1.0 |
| PXD057215 (Anammox reactor) | 1.6 | 0.9 | 0.3 | 0.3 |
| PXD024291 (Human gut model) | 94.4 | 37.4 | 1.5 | 0.5 |

Supplementary Table S8: Overview of analyzed datasets

| **Dataset** | **Identifier** | **Files analyzed by MetaProSIP workflow** | **Analysis deposited to** |
| --- | --- | --- | --- |
| Standard *Escherichia coli* K12 cultures cultivated with 1.07–99% ^13^C [32] | PXD041414 | none | [10.5281/zenodo.15537146](https://doi.org/10.5281/zenodo.15537146) |
| Mock community with spike-in of ^13^C-labeled *Escherichia coli* [27] | PXD024174 | Run1_MockU2_EcoliR1_10_2000ng.raw  Run1_MockU2_EcoliR2_10_2000ng.raw  Run1_MockU2_EcoliR3_10_2000ng.raw | [10.5281/zenodo.16317854](https://doi.org/10.5281/zenodo.16317854) |
| ‘*Candidatus* Kuenenia stuttgartiensis’ strain CSTR1 reactor | PXD057215 | 01_6_27_12C_01.raw  02_6_27_12C_02.raw  03_6_27_12C_03.raw  19_7_01_mix_01.raw  20_7_01_mix_02.raw  21_7_01_mix_03.raw  22_7_18_mix_01.raw  23_7_18_mix_02.raw  24_7_18_mix_03.raw | [10.5281/zenodo.16318298](https://doi.org/10.5281/zenodo.16318298) |
| Model of the human distal gut microbiome [14, 27] | PXD024291 | 2017-02-07_RS_Robo_9.raw  2017-02-07_RS_Robo_10.raw  2017-02-07_RS_Robo_11.raw  2017-02-07_RS_Robo_12.raw  2017-02-07_RS_Robo_13.raw  2017-02-07_RS_Robo_14.raw  2017-02-07_RS_Robo_15.raw  2017-02-07_RS_Robo_16.raw  2017-02-07_RS_Robo_17.raw  2017-02-07_RS_Robo_18.raw  2017-02-07_RS_Robo_19.raw  2017-02-07_RS_Robo_20.raw | [10.5281/zenodo.16318031](https://doi.org/10.5281/zenodo.16318031) |


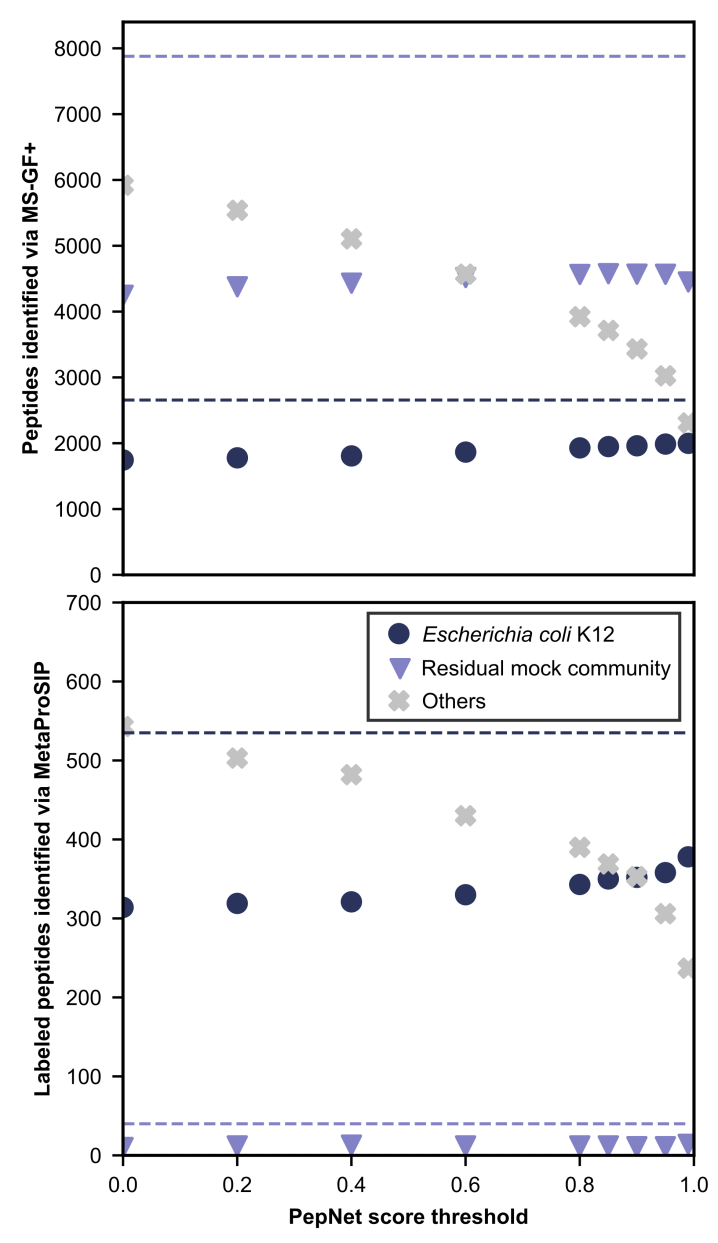


Supplementary Figure S1: Comparison of unlabeled and ¹³C-labeled peptide identifications in a mock microbial community spiked with ^13^C-labeled *Escherichia coli* K12 cells using MS-GF+ and MetaProSIP with *de novo* peptide databases (1% PSM-level FDR) and a metagenome-derived protein database (2% PSM-level FDR). Raw data was obtained from [27]. The score threshold of >0.99 is highlighted in yellow. Only peptides detected in at least two samples are plotted. **A**: *De novo* peptide databases were generated using Pepnet and filtered at varying quality score thresholds. Identified peptides are color-coded based on their presence in the E. coli K12 reference protein database (PRIDE accession number: PXD024285) or common contaminants (navy circles), the metagenome-derived database of the residual mock community (PRIDE accession number: PXD006118, purple triangles), or neither (grey crosses). Dashed lines represent identifications from the metagenome-derived database.


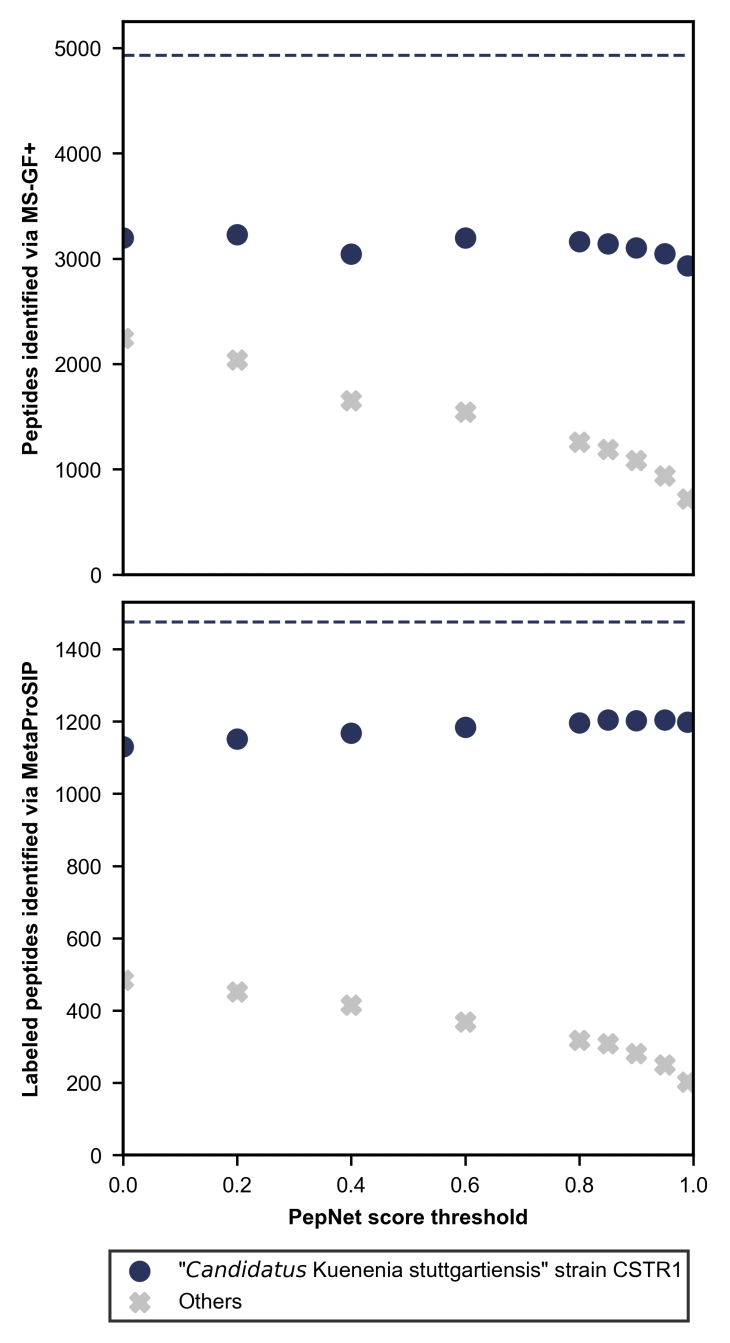


Supplementary Figure S2: Comparison of unlabeled and ¹³C-labeled peptide identifications using MS-GF+ and MetaProSIP in an enrichment of ‘*Candidatus* Kuenenia stuttgartiensis’ strain CSTR1 from a continuous flow reactor fed with ^13^C-bicarbonate with *de novo* peptide databases (1% PSM-level FDR) and a genome-derived protein database (2% PSM-level FDR). The score threshold of >0.99 is highlighted in yellow. Only peptides detected in at least two samples are plotted. **A**: *De novo* peptide databases were generated using PepNet and filtered at varying quality score thresholds. Navy circles represent peptide identifications matching the genome-derived database (NCBI: CP049055.1) or universal contaminants; grey crosses represent unmatched peptides. Dashed lines show identification counts using the genome-derived database.


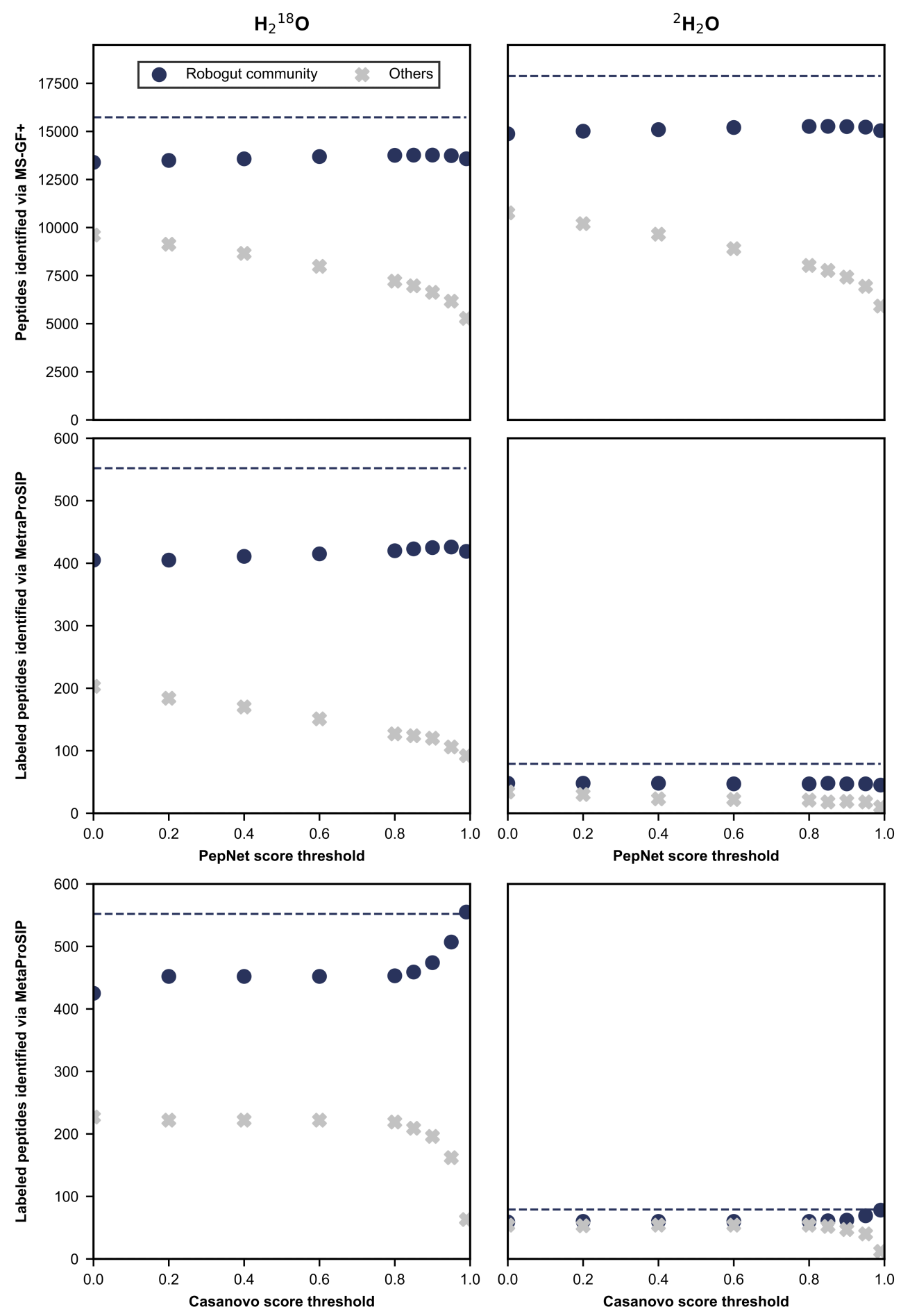


Supplementary Figure S3: Comparing *de novo* peptide databases for identifying unlabeled and ^2^H/^18^O-labeled peptides via MS-GF+ and MetaProSIP (1% PSM-level FDR) in a model of the human gut microbiome (Robogut community) cultivated with ^2^H_2_O or H_2_^18^O, respectively, and obtained from [14]. *De novo* peptide databases were assembled using PepNet or Casanovo and filtering identifications with different thresholds for the quality score. Navy circles denote the number of identified peptides present in the genome-derived reference database or universal contaminants. Grey crosses denote the number of identified peptides absent in the genome-derived reference database and universal contaminants. Dashed lines represent the number of peptides identified using the genome-derived reference database appended with universal contaminants. Only peptides detected in at least two samples are plotted.


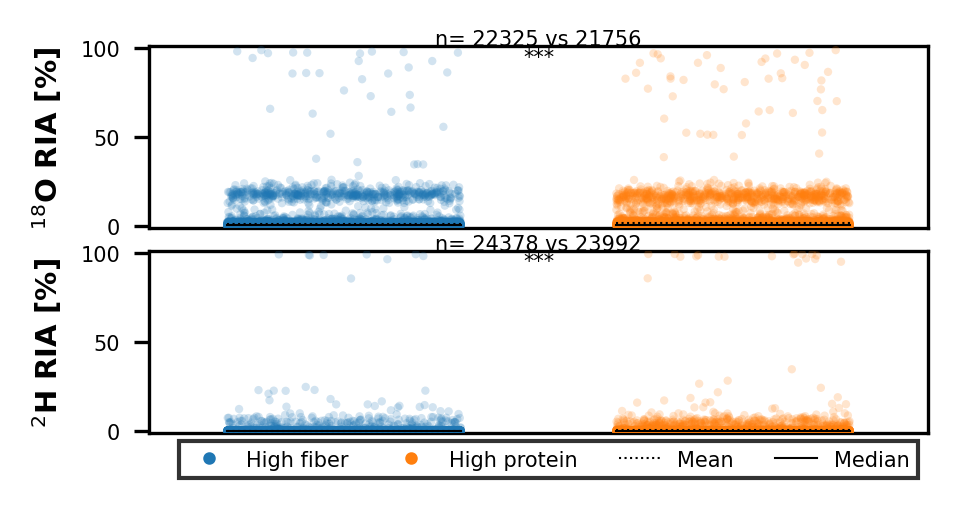


Supplementary Figure S4: Distribution of the ^18^O and ^2^H relative isotope abundance (RIA) in detected peptides identified in a model of the human gut microbiome cultivated in either a high fiber or high protein medium in the presence of either H_2_^18^O or ^2^H_2_O. RIA values are shown for peptides detected in at least two replicates of both media. ‘n’ denotes the number of peptides identified across triplicates. Statistically significant differences in RIA between media are indicated with ‘***’ based on Student's t-test for the means of two independent samples with p < 0.001.


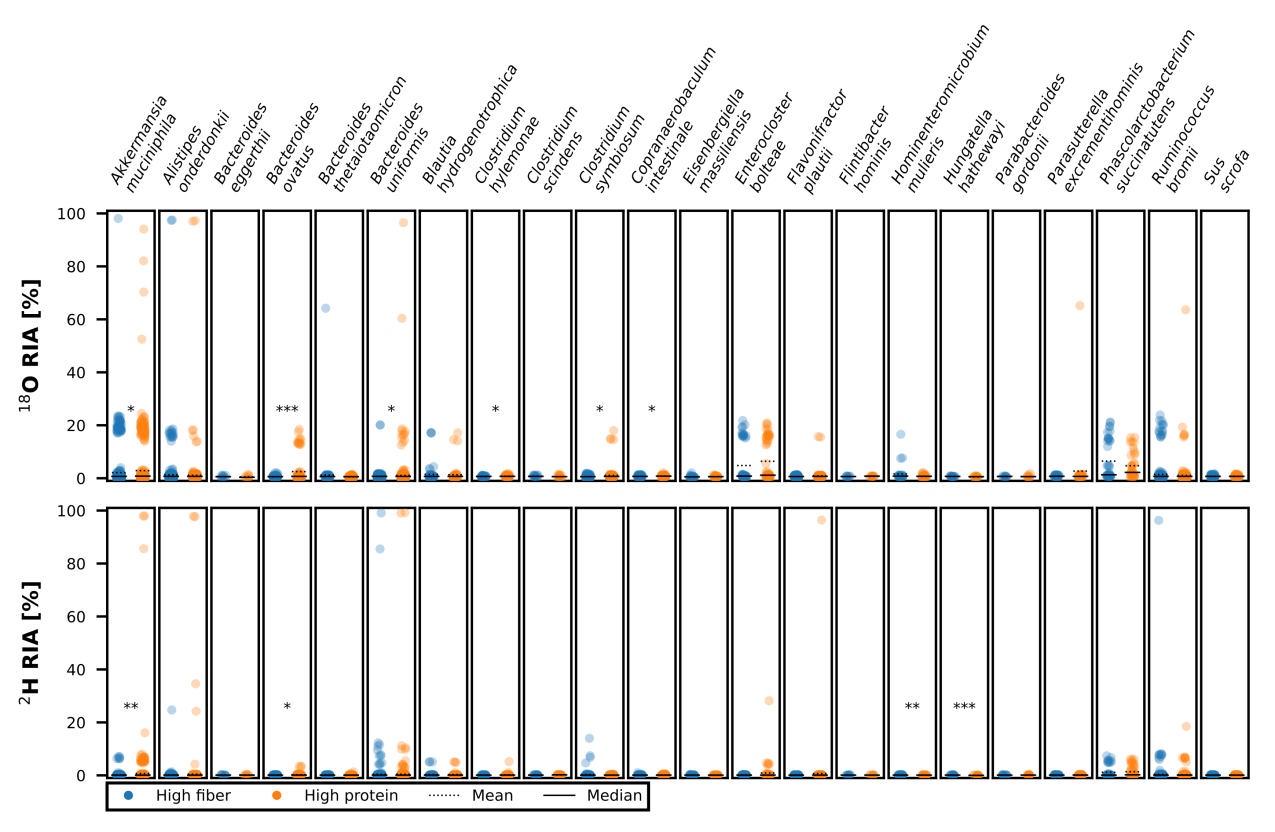


Supplementary Figure S5: Figure 8 without removal of outliers.


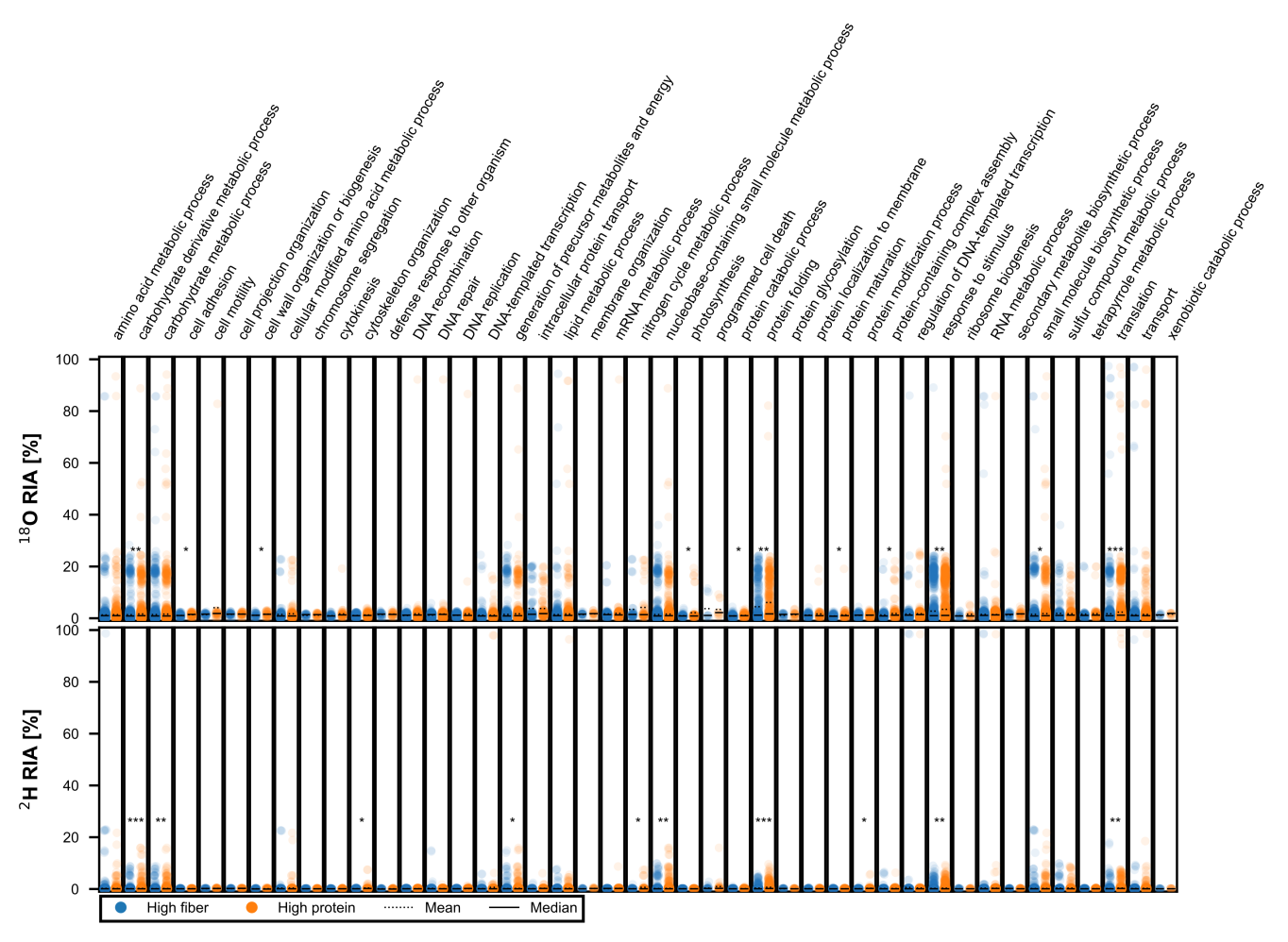


Supplementary Figure S6: Figure 9 without removal of outliers.

## Investigation of peptides categorized as ‚others’ in Figures 3, 5, and 7.

To investigate the origin of peptides not covered by the reference databases, and consequently categorized as ‘other’ in Figures 3, 5, and 7, we analyzed all peptides identified by MS-GF+ with CS > 0.99 that were detected in at least two samples per dataset (Supplementary Table S9). These ‘other’ peptides were absent from the genome-based reference databases but were further classified based on whether they could be found in UniProt via Unipept.

Supplementary Table S9: Number of peptides identified via MS-GF+ with the CS>0.99 databases in at least two samples per dataset. Peptides absent in the genome-based reference database are labeled as ‘other’. ‘Other’ peptides are further divided based on whether they are found in UniProt 2025.03 via Unipept 6.2.5.

| **Dataset identifier** | | Total peptides identified | Other peptides  (% of total) | Other peptides in UniProt  (% of other) | Other peptides not in UniProt  (% of other) |
| --- | --- | --- | --- | --- | --- |
| **PXD024174**  **(Mock community)** | | 8,493 | 687  (8.1%) | 147  (21.4%) | 540  (78.6%) |
| **PXD057215**  **(Anammox reactor)** | | 3,842 | 323  (8.4%) | 43  (13.3%) | 280  (86.7%) |
| **PXD024291**  **(Human gut model)** | ^18^O | 20,450 | 4,158  (20.3%) | 2,510  (60.4%) | 1,648  (39.6%) |
|  | ^2^H | 23,316 | 4,922  (21.1%) | 2,914  (59.2%) | 2,008  (40.8%) |

In the mock community and anammox reactor datasets, only a small proportion of peptides (8.1% and 8.4%, respectively) were not found in the reference databases, which were generated from sample-matching metagenomes. Of these ‘other’ peptides, a subset (21.4% in the mock community and 13.3% in the anammox reactor) was found in UniProt and largely affiliated with expected taxa, such as *Chlamydomonas reinhardtii*, *Salmonella enterica*, and *Paracoccus pantotrophus* in the mock community, or *Pseudomonadota* and *Eukaryota* in the anammox reactor.

In contrast, the **human gut model** datasets showed a substantially higher proportion of peptides absent from the reference database, 20.3% and 21.1% in the ^18^O and ^2^H datasets, respectively. However, most of these peptides were present in Uniprot and thus likely belonging to organisms not included by the reference database. This reflects inherent limitations of the reference database, which was constructed from public proteomes of cultured isolates identified via 16*S* rRNA gene amplicon sequencing. As a result, strain-level diversity, sequence variants, or uncultured taxa may not be represented. Notably, our analysis uncovered peptides associated with two species that were entirely absent from the reference database, further underscoring its incompleteness.

To further investigate the peptides absent from both the reference and UniProt, we performed local BLASTP (version 2.16.0) searches on the Galaxy [92, 102] instance of the Helmholtz Centre for Environmental Research – UFZ: (1) against the genome-derived reference databases, and (2) against NCBInr (database version: 2025-03-31). The top hits (lowest E-value) for each peptide sequence were examined (Supplementary Figure S7).


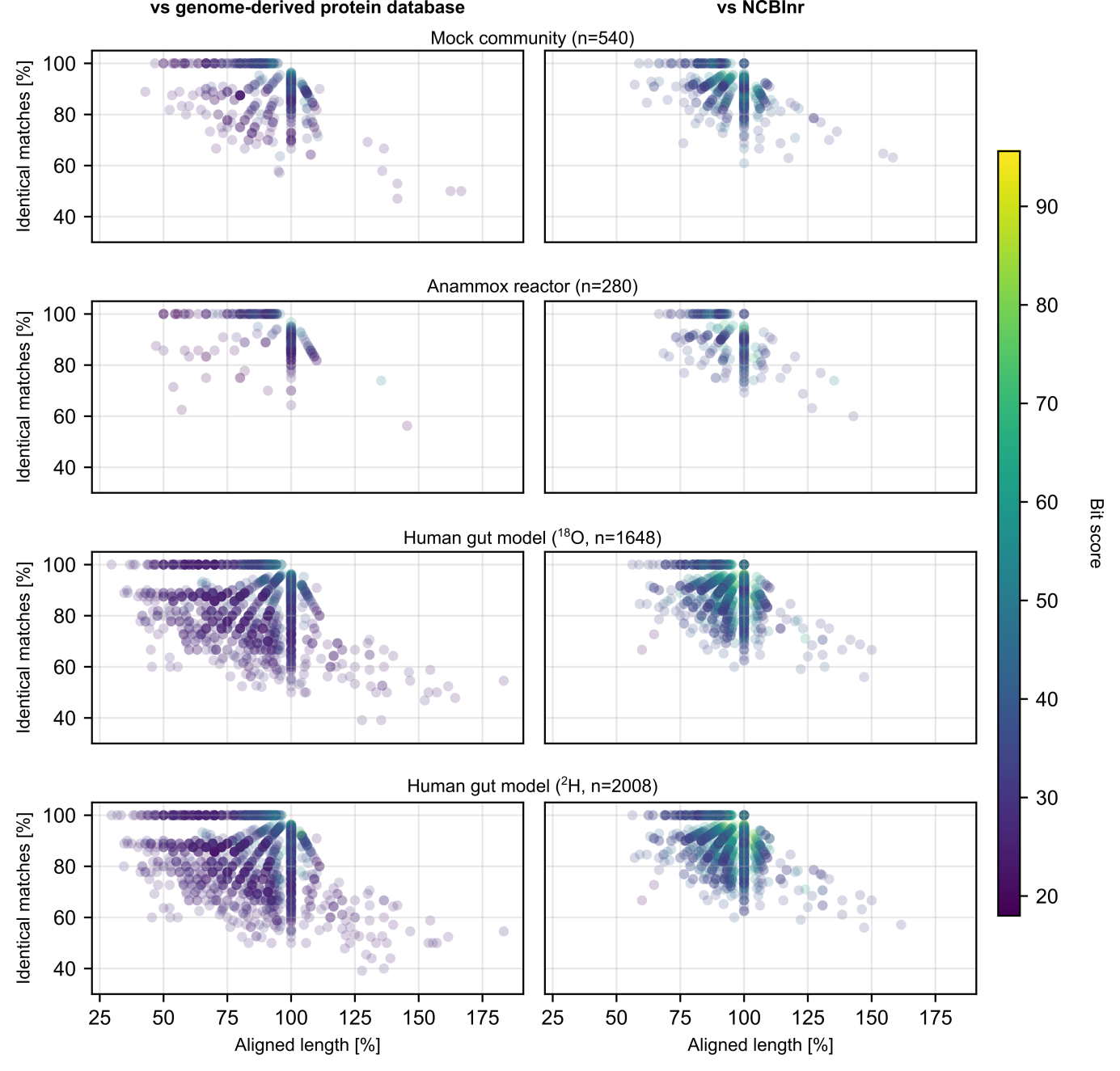


Supplementary Figure S7: BLASTP of peptides identified with the CS>0.99 *de novo* peptide database but absent from both the genome-derived protein database and UniProt. Aligned length was calculated as: $\text{ }\frac{\text{alignment length}}{\text{query sequence length}}\text{∙100\%}$. Only the best hits (lowest E-value) are plotted.

Many peptides identified with the CS>0.99 *de novo* peptide database but absent from both the genome-derived protein database and UniProt showed high sequence similarity to known peptides in the genome-derived protein database, differing by only a single amino acid substitution or inversion of adjacent amino acids, which are considered as common *de novo* prediction errors [103].

Using the anammox reactor dataset as a representative example due to its lower complexity, we found that 134 out of 280 peptides not present in the genome-derived protein database or UniProt matched best to taxa in NCBInr expected in the sample (*e.g.,* anammox taxa or *Pseudomonadota*). The remaining 146 peptides were predominantly associated with a wide range of taxa, largely represented by only a single hit each.

This raises an open question in the metaproteomics field: how should we interpret *de novo* predicted peptides that are highly similar, but not identical, to expected sequences? Their presence may reflect true biological variation (*e.g.*, mutations), genome sequencing or assembly errors in reference databases, or point errors from *de novo* peptide prediction. To validate these peptides and distinguish true novel identifications from errors, orthogonal approaches such as ribosome profiling (Ribo-seq) or metatranscriptomics would be essential [81, 104].
